# Petechiae and Desquamation of Fingers Following Immunization With BTN162b2 Messenger RNA (mRNA) COVID-19 Vaccine

**DOI:** 10.7759/cureus.16858

**Published:** 2021-08-03

**Authors:** Nathaniel J Irvine, Brittany L Wiles

**Affiliations:** 1 Family Medicine, Naval Hospital Jacksonville, Jacksonville, USA

**Keywords:** covid19, pfizer-biontech vaccine, petechiae, desquamation, adverse reactions, covid-19 vaccine

## Abstract

Since late 2019, the coronavirus disease 2019 (COVID-19) pandemic, caused by severe acute respiratory syndrome coronavirus2* *(SARS-CoV-2), has killed over three million people. More than 600,000 of these deaths have occurred in the United States alone. While advances in the treatment of COVID-19 have been made, the advent of highly effective vaccines against this coronavirus variant has given hope that the end of the pandemic may be near. Unfortunately, resistance towards vaccination remains a barrier to virus eradication both in the United States and globally. The driving factor for much of this opposition is the concern over potential adverse reactions from the vaccines against SARS-CoV-2. In order to combat this, it is imperative that vaccine side effects and their corresponding clinical course are clearly described. This report details the case of a female patient who developed acral petechiae, desquamation of the fingers, and a facial rash that occurred shortly after receiving the second dose of the Pfizer-BioNTech COVID-19 vaccine.

## Introduction

The coronavirus disease 2019 (COVID-19) pandemic, found to be caused by the highly infectious coronavirus severe acute respiratory syndrome coronavirus 2 (SARS-CoV-2), has become a global health and economic crisis since it first emerged in 2019 [1]. Initially, there were limited prevention and treatment options, but by the end of 2020, the United States Food and Drug Administration had approved two vaccines for emergency use [2]. There has been widespread hesitation amongst the global population in several countries to receive the vaccine. Therefore, it is important for all potential side effects to be reported for the purpose of transparency and to assess the clinical course of these side effects. Herein we report a case of acral petechiae and finger desquamation accompanied by a facial rash occurring shortly after vaccination with the Pfizer-BioNTech vaccine.

## Case presentation

A 43-year-old black female presented to the clinic after being evaluated in the emergency department for new-onset acral petechiae and a facial rash. Her past medical history was notable for obstructive sleep apnea, hypertension, migraines, type 2 diabetes, and obesity. Her medication regimen consisted of chlorthalidone, amlodipine, amitriptyline, and sitagliptin. She had been prescribed and was taking these medications for several years. The patient’s only reported drug allergy was a rash precipitated by amoxicillin when she was a child. Her history was negative for any recent travel or sick contacts; however, she had received the second dose of the Pfizer-BioNTech COVID-19 vaccine a few days before the onset of her symptoms. She denied any side effects from the first vaccine dose, but a day after receiving her second dose, she developed fatigue and myalgias. The fatigue and myalgias improved over the following days, but five days after receiving the second dose, the petechiae and rash developed.

**Figure 1:**
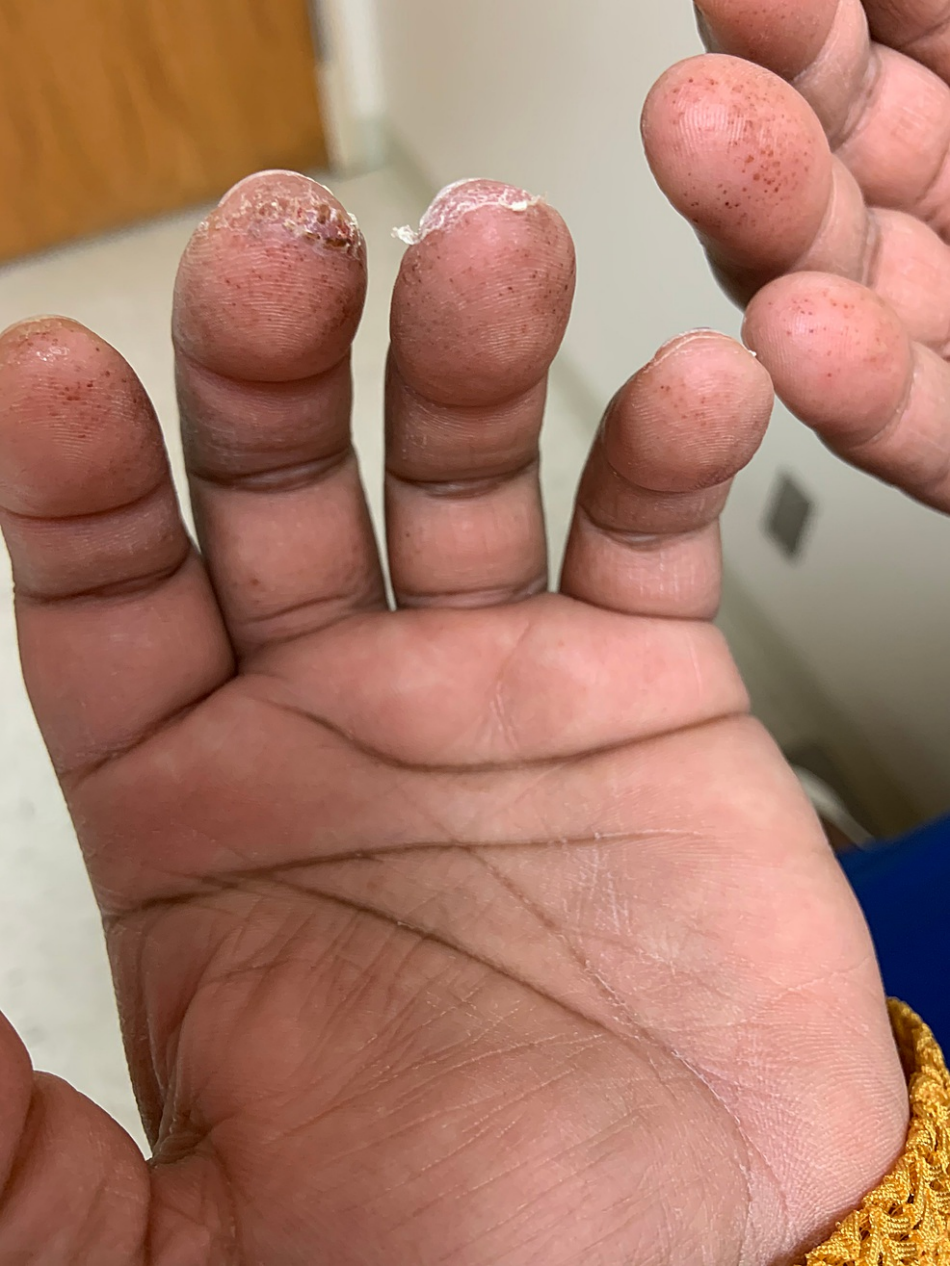
Petechiae and desquamation of patient's fingers

**Figure 2:**
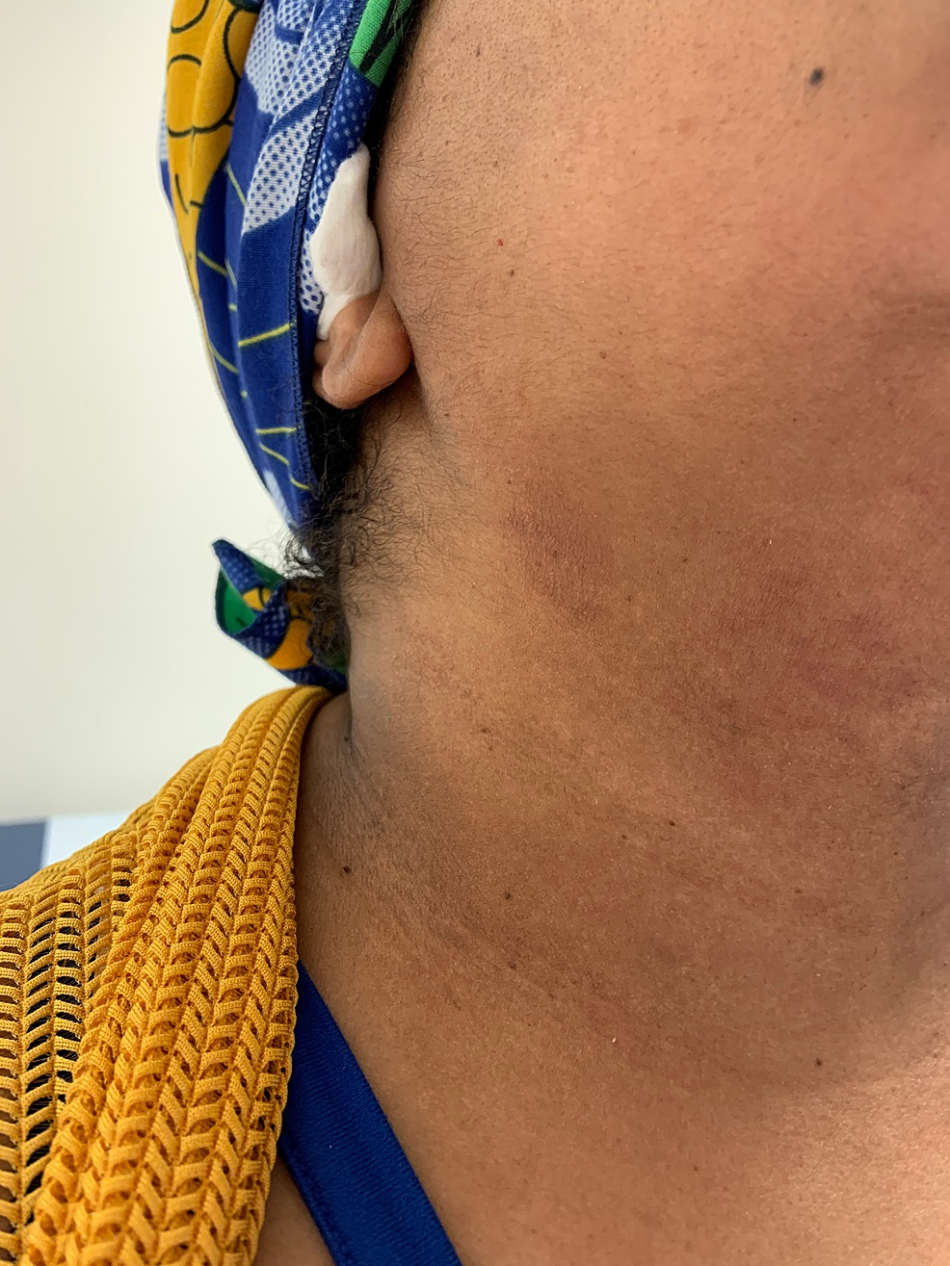
Patient's facial rash

The physical examination was notable for petechiae and desquamation on the distal aspects of her fingers (Figure 1). A hyperpigmented, non-blanching, morbilliform, and macular rash was present on the patient’s jawline on the right side of her face (Figure 2). An extensive laboratory evaluation was performed. A complete blood count, basic metabolic panel, and urinalysis were unremarkable. A fibrin D-dimer was elevated at 0.62, but other coagulation studies were within normal limits. Rheumatologic labs, including antinuclear antibodies, antineutrophil cytoplasmic antibodies, anti-cyclic citrullinated peptide, anti-Ro/SSA, anti-La/SSB, antiribonucleoprotein, anti-Smith, anti-dsDNA, anti-cardiolipin, anti-histone, anti-beta-2-glycoprotein I, and rheumatoid factor, were all negative. A polymerase chain reaction for SARS-CoV-2 was negative. Of note, complement components C3 and C4 were elevated at 197 mg/dL and 46 mg/dL, respectively. The erythrocyte sedimentation rate and C-reactive protein were also high at 43 mm/hr and 11.0 mg/dL, respectively.

As the patient was clinically stable and the etiology uncertain, she was managed with close interval follow-up. At her one-week follow-up, her complete blood count remained the same, with no evidence of thrombocytopenia. The number of petechiae on her fingers had increased and the desquamation slightly worsened. Two weeks later, the patient reported full resolution of her symptoms.

## Discussion

Since the COVID-19 pandemic first began in 2019, it has spread across the globe, affecting millions. It is highly transmissible through respiratory secretions such as coughing and even talking. Indoors, the virus can remain as an aerosol and remain highly infectious [3]. The virus is thought to enter cells via the angiotensin-converting enzyme 2 receptor that is present in the mucosal cells of the mouth, pneumocytes of the alveoli, and endothelial cells [4]. Both innate and adaptive immune responses are triggered, which in most cases leads to an eradication of the virus. However, in some severe cases, there is a rise in inflammatory cytokines, which along with direct damage from the virus results in lymphopenia. Consequently, there is a dysregulated immune response that is ineffective at clearing the virus leading to pneumonia and acute respiratory distress syndrome [5]. By the end of March 2021, more than 2.7 million people worldwide were confirmed dead with or from COVID-19, with 20% of those deaths occurring in the United States. If the pandemic were to continue unchecked, it is estimated that $1.4 trillion would be lost from the United States’ gross domestic product [6]. This, along with the high death toll, demonstrates the need to prevent COVID-19 and its complications.

In December 2020, the United States Food and Drug Administration approved two vaccines to prevent disease. The first, the BTN162b2 mRNA COVID-19 vaccine, was developed by Pfizer-BioNTech, and the second, the mRNA-1273 SARS-CoV-2 vaccine, by Moderna [3]. A two-dose regimen of the Pfizer-BioNTech vaccine was found to be 95% effective at preventing symptomatic infection [2]. Due to the fact these vaccines used nucleic acid-based technology, there was an unexpected hesitancy amongst many to get vaccinated [7]. Also, there was a concern that the vaccines were rushed through development. Survey data from the United States showed 27% of individuals were hesitant to receive a vaccine against COVID-19 due to potential adverse reactions. A majority of the adverse reactions reported since the development of these vaccines have been local cutaneous reactions [8].

A registry analysis of COVID-19 vaccine cutaneous reactions found 414 patients between December 2020 and February 2021 who reported reactions to either the Pfizer-BioNTech or the Moderna COVID-19 vaccine. Most reactions were local injection site reactions, including swelling, erythema, and pain. A registry analysis of COVID-19 vaccine cutaneous reactions by McMahon et al. showed 20% of the patients who received the Pfizer-BioNTech COVID-19 vaccine reported reactions after receiving their first vaccine dose. After the second dose, 74% of patients reported cutaneous manifestations. The remaining 6% had reactions associated with both doses [9]. None of the patients in the above registry analysis had acral petechiae associated with either vaccine.

The differential causes for primary acral petechiae include inflammatory processes, embolism, infection, trauma, and vasculitis [10]. Dermatitis herpetiformis is an inflammatory condition that could cause petechiae, but our patient had no associated vesiculopapular lesions, gastrointestinal symptoms, or a history of celiac disease. An embolic source was thought to be unlikely given the lack of symptoms and exam findings. The patient had no evidence of an infection and there was no history of trauma. A vasculitis was thought to be the cause of our patient’s symptoms; however, aside from elevated inflammatory markers, the laboratory workup was unrevealing. While SARS-CoV-2 infection is known to cause microangiopathic injury and thrombosis via activation of the complement system, our patient’s complement components C3 and C4 were slightly elevated [5]. Given the temporal relationship to her vaccination, it is likely that the inflammatory process associated with vaccination caused a transient vasculitis, which led to the petechiae. As the inflammation subsided post-vaccination, so too did the petechiae. It is interesting that in addition to the acral petechiae, she had desquamation on the tips of her fingers. This has been seen both with COVID-19 infections and vaccines as have morbilliform rashes like the one on her face [8,11].

## Conclusions

This case report characterizes a previously unknown reaction to the Pfizer-BioNTech COVID-19 vaccine that, to the best of our knowledge, has not been reported. Like previously reported cutaneous reactions to the Pfizer-BioNTech COVID-19 vaccine, our patient’s course was self-limited and resolved without intervention. Given the importance of continued global vaccination against the SARS-CoV-2 virus, it is imperative that these vaccine reactions are well-characterized allowing clinicians and their patients to make informed decisions and prevent vaccine resistance for benign side effects.
